# Supplementary material for: A Flexible Sensor with Excellent Environmental Stability Using Well-Designed Encapsulation Structure
Source: Polymers (Basel). 2023 May 15;15(10):2308. doi: 10.3390/polym15102308 (PMC10224181; doi:10.3390/polym15102308)
Supplement: Supplementary file 1 [file polymers-15-02308-s001.zip › polymers-2383668-supplementary.pdf]

Supporting Information

A Flexible Sensor with Excellent Environmental Stability Using Well-Designed Encapsulation Structure

Jian Zou [1,2,†], Zhuo Chen [1,2,†], Sheng-Ji Wang [1,2], Zi-Hao Liu [1], Yue-Jun Liu [1,2], Pei-Yong Feng [2] and Xin Jing [1,2,*]

1 Key Laboratory of Advanced Packaging Materials and Technology of Hunan Province, Hunan University of Technology, Zhuzhou 412007, China

2 National and Local Joint Engineering Research Center for Advanced Packaging Material and Technology, Hunan University of Technology, Zhuzhou 412007, China

* Correspondence: jingxin@hut.edu.cn

† These authors contributed equally to this work.

Corresponding Authors:

Xin Jing E-mail: jingxin@hut.edu.cn

# Jian Zou and Zhuo Chen contributed equally to this work.

Note:

The authors declare no competing financial interest.

This supporting information contains:

pages (S1-S8)

figures (Figure S1-S9)

1. The encapsulation mold

In order to fully encapsulate the cylindrical hydrogel, a special sleeve and cover molds were printed using a fused deposition 3D printer (CP-01, Shenzhen Chuangxiang 3D Technology Co., Ltd.) with polylactic acid wires (Figures S1a and S1b). The inner diameter of the hollow tube part of lower sleeve and upper cover is 17 mm and 20.5 mm, respectively, and their heights are both 18 mm. Figure S1c is a schematic diagram of the assembled mold. The printing speed was 50mm/s, the temperature of the print head and printing platform was 200 °C and 45 °C, respectively. In order to prevent the mold from shifting during the printing process, masking tape was laid on the printing platform to increase friction and improve the printing accuracy.

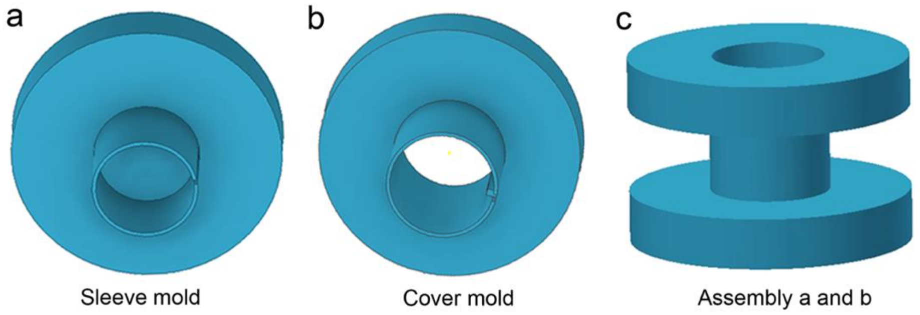

Figure S1. (a) Schematic diagrams of upper cover; (b) Schematic diagrams of lower sleeve; (c) Schematic diagrams of the assembled mold

2. lap-shear test

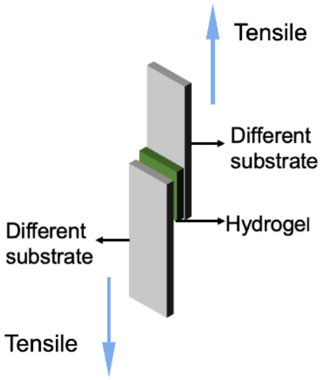

Figure S2. Scheme illustration of the lap-shear test

3. long-term cyclic step strain test

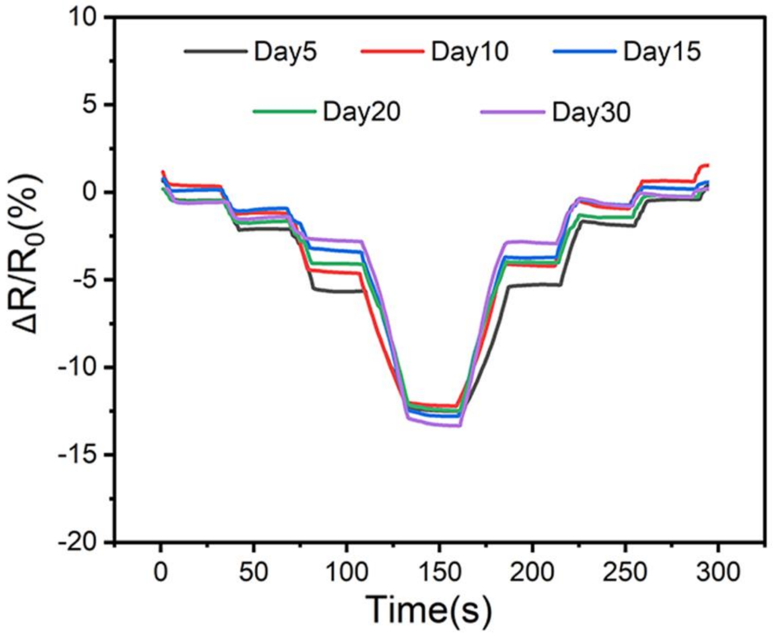

Figure S3. The relative resistance change of the hydrogel-based sensor in the cyclic step strain tests for different periods

4.

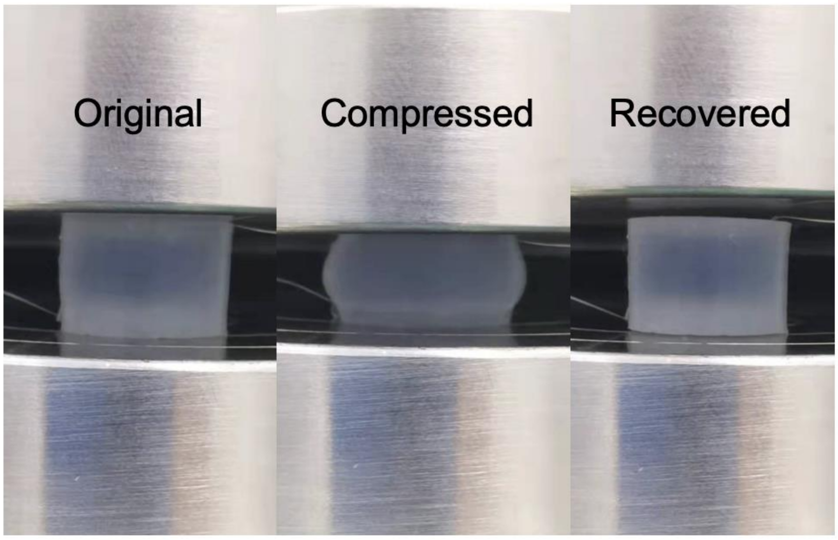

Figure S4. The image of the assembled hydrogel-based sensor during loading- unloading process

5. The output signals of the hydrogel-based sensor before and after encapsulation upon different compression speeds.

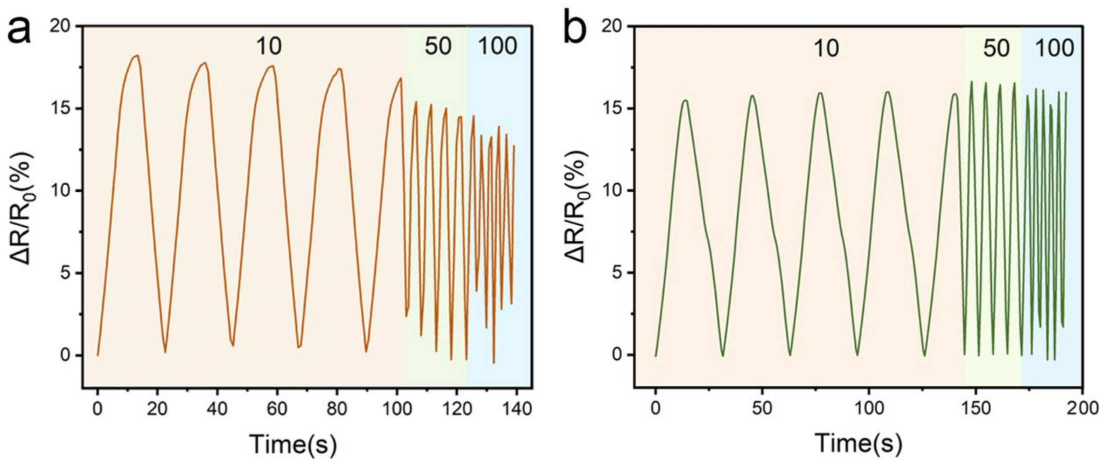

Figure S5. (a) The relative resistance change of the the hydrogel-based sensor before encapsulation upon cyclic compression under different compression speeds; (b) The relative resistance change of the hydrogel-based sensor after encapsulation upon cyclic compression under different compression speeds

6. Hydrogel-based sensor with conductive carbon tape as electrode

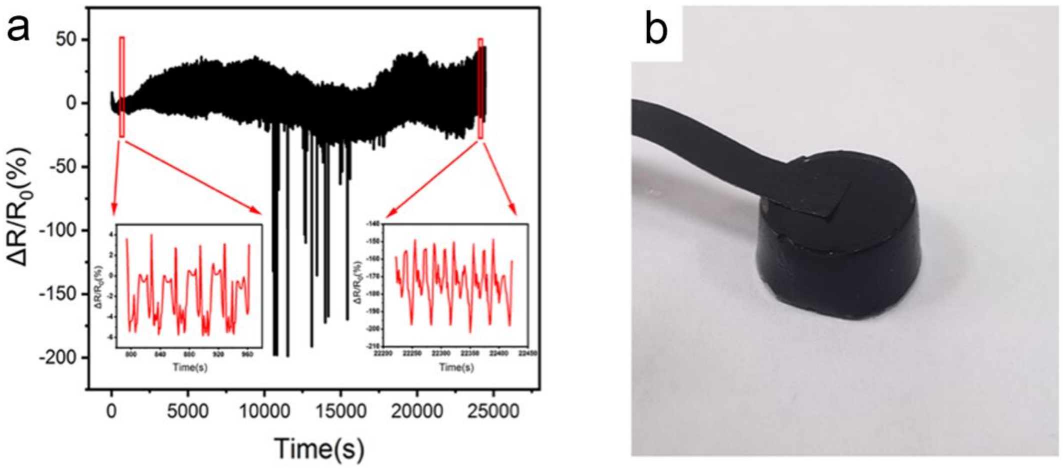

Figure S6. (a) Relative resistance change of the hydrogel-based sensor with conductive carbon tape as electrodes; (b) The connection between conductive carbon tape and hydrogel

7. The 3D depth-of-field image of cylindrical hydrogels

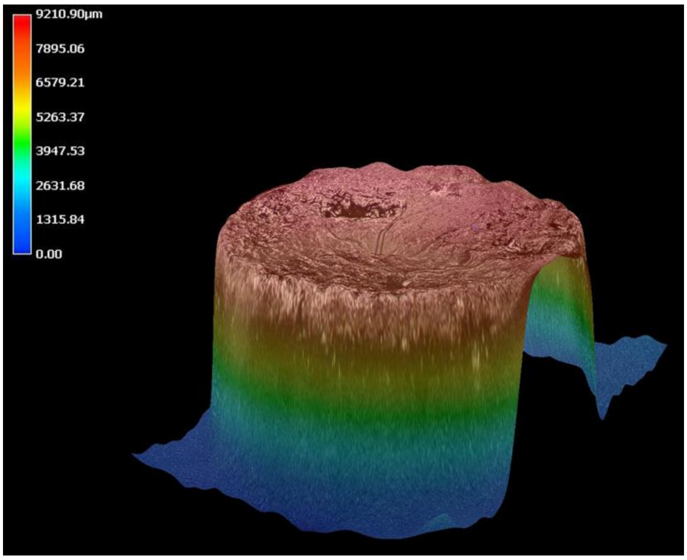

Figure S7. The 3D depth-of-field image of cylindrical hydrogels

8. The establishment of the simulation analysis model

The standard/explicit module of Abaqus was used to build and analyze the model. The overall size of the hydrogel and Ecoflex layer in the model were consistent with the actual size of the encapsulated hydrogel. Figures S8a and S8b are the model images after "view cut" operation from the X-plane and Z-Plane planes, respectively. It can be seen that the hydrogel was completely encapsulated in the Ecoflex, and the electrodes were placed on the upper and lower surfaces of the hydrogel. For the material property setting, the Young's modulus of the hydrogel, Ecoflex, and nickel-chromium wire were set as 0.012 MPa, 0.2 MPa, and 2240 GPa, respectively, their Poisson's ratios were set as 0.49, 0.45, and 0.3, respectively. The Poisson's ratio of nickel chromium wire was obtained by querying the manufacturer. In order to obtain the Poisson's ratio of hydrogel and Ecoflex, an electronic universal testing machine was employed to compress the prepared cylindrical hydrogel and Ecoflex to 20% strain, and then the transverse strain was measured by Vernier caliper. Finally, Poisson's ratio was calculated by the transverse strain and the strain in the compression direction. For the boundary condition setting, as shown in Figure S8d, a boundary condition (U1= U2= U3= UR1= UR2= UR3= 0)

was created on the lower surface of the model. A vertical load was applied to the upper surface of the model, resulting in a 20% strain in the model. Eight-node linear hexahedral element (C3D8) was selected as the three-dimensional finite element model of hydrogel and Ecoflex; Two-node linear three-dimensional truss element (T3D2) was selected as the three-dimensional finite element model of nickel-chromium wire. The approximate global size was set to 0.5, and the maximum deviation factor was 0.1. The meshing result is shown in Figure S8c and 8e.

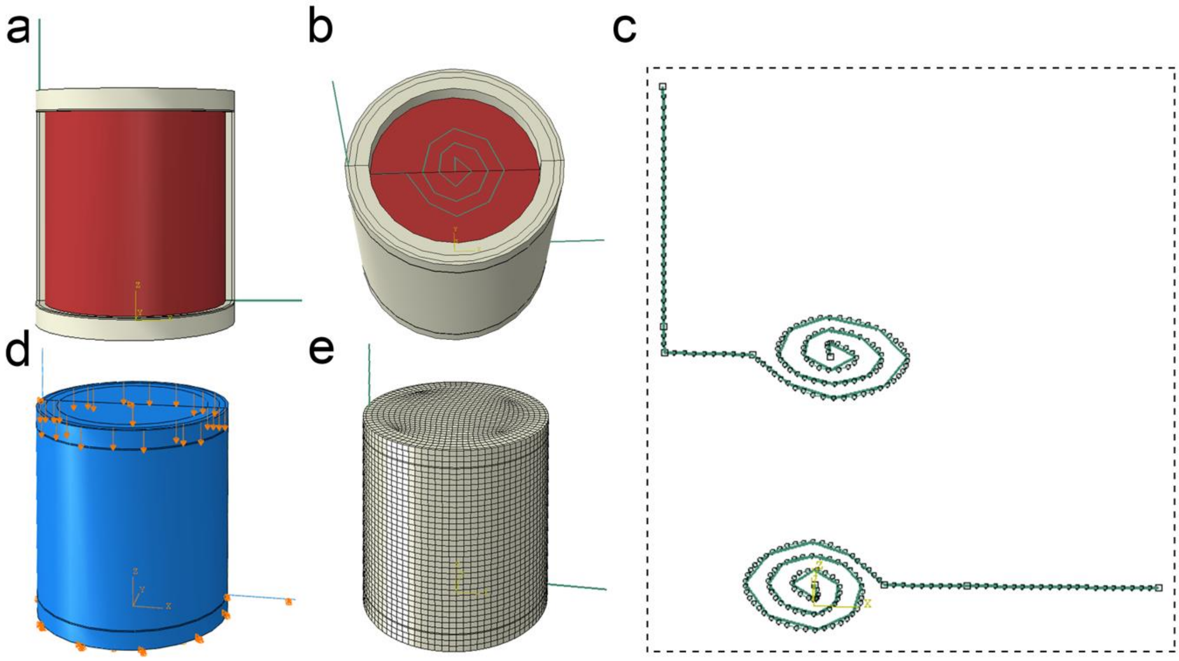

Figure S8. (a) The image of the model after “view cut” operation from the X-plane; (b) The image of the model after “view cut” operation from the Z- plane; (c) Schematic diagram of meshing of electrodes; (d) Schematic diagram of boundary conditions; (e) Schematic diagram of meshing of hydrogel and Ecoflex

9. The comparaion of the theoretical and simulated values

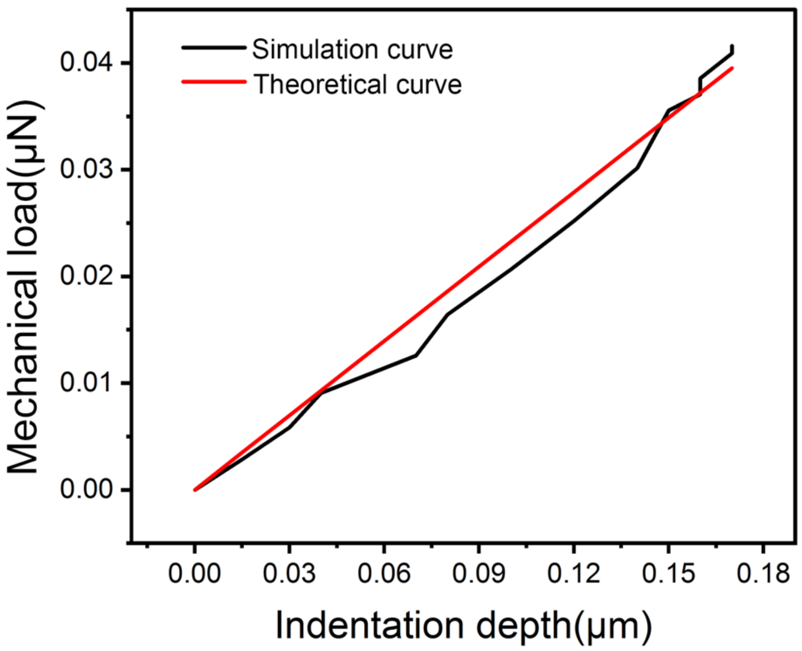

Figure S9. The curves of the Load-Depth obtained from simulation and theoretical analysis
